# Experimental evidence of inbreeding depression for competitive ability and its population-level consequences in a mixed-mating plant

**DOI:** 10.3389/fpls.2024.1398060

**Published:** 2024-09-17

**Authors:** Mark J. Walker, Rachel B. Spigler

**Affiliations:** Department of Biology, Temple University, Philadelphia, PA, United States

**Keywords:** inbreeding depression, asymmetric competition, soft selection, density-dependence, mating system evolution, mutation load, competitive ability, ecological neighborhood

## Abstract

Inbreeding depression is a key factor regulating the evolution of self-fertilization in plants. Despite predictions that inbreeding depression should evolve with selfing rates as deleterious alleles are increasingly exposed and removed by selection, evidence of purging the genetic load in wild populations is equivocal at best. This discordance could be explained, in part, if the load underlying inbreeding depression is subject to soft selection, i.e., the fitness of selfed individuals depends on the frequency and density of selfed vs. outcrossed individuals in the population. Somewhat counterintuitively, this means that populations with contrasting mutation load can have similar fitness. Soft selection against selfed individuals may be expected when there is inbreeding depression for competitive ability in density-regulated populations. We tested population-level predictions of inbreeding depression in competitive ability by creating a density series of potted plants consisting of either purely outcrossed, purely selfed, or mixed (50% outcrossed, 50% selfed) seed of the mixed-mating biennial *Sabatia angularis* (Gentianaceae) representing ecological neighborhoods. Focusing on the growth and survival of juveniles, we show that mean plant size is independent of neighborhood composition when resources are limiting, but greatest in outcrossed neighborhoods at low densities. Across a range of densities, this manifests as stronger density-dependence in outcrossed populations compared to selfed or mixed ones. We also found significantly greater size inequalities among individuals in mixed neighborhoods, even at high densities where mean juvenile size converged, a key signature of asymmetric competition between outcrossed and selfed individuals. Our work illustrates how soft selection could shelter the genetic load underlying inbreeding depression and its demographic consequences.

## 1 Introduction

Questions about the factors that regulate population growth and drive natural selection are among the most basic in ecology and evolution. Although the idea that these two processes intersect is not inherently novel (e.g., Lande, 1982; Bassar et al., 2021), explicit links are often overlooked. For example, ecological models of density dependence typically assume all genotypes within a population are equally impacted by density, while many evolutionary models assume an individual’s absolute fitness is a global parameter, independent of ecological context and determined solely by its genotype (i.e., selection is ‘hard’, sensu Wallace, 1975 and Bell et al., 2021). Although the latter will apply to lethal mutations, the fitness effects of other genes may be determined locally, dependent on an individual’s own genetic quality relative to the local average (i.e., selection may be ‘soft’; Agrawal, 2010; Agrawal and Whitlock, 2012; Bell et al., 2021). In density-regulated populations, soft selection could arise when genotypes differ in competitive ability. From an ecological perspective, this means that, all else equal, the strength of density dependence and the demographic impacts of selection may vary with the genetic composition of a population. Making these connections is important, because when density-dependent population regulation is combined with genetic variation in competitive abilities, opportunities for ‘eco-genetic’ feedbacks can arise (Antonovics and Levin, 1980; Kokko and López-Sepulcre, 2007; Metcalf and Pavard, 2007; Shefferson and Salguero-Gómez, 2015; Bassar et al., 2021), providing possible answers to longstanding questions in evolutionary biology such as the maintenance of mutation load and polymorphisms in populations.

Inbreeding depression—the reduced fitness of offspring produced via inbreeding compared to those produced via outcrossing—is often used in models to illustrate the potential for soft selection and its evolutionary and ecological consequences (Wallace, 1975; Whitlock, 2002; Agrawal, 2010). Moreover, inbreeding depression is considered the key element regulating the evolution of self-fertilization (“selfing”) in hermaphroditic organisms (Jarne and Charlesworth, 1993). Inbreeding depression is a manifestation of mutation load in populations, thought to be caused primarily by the expression of deleterious recessive alleles in homozygous individuals (Charlesworth and Charlesworth, 1999; Charlesworth and Willis, 2009; Brown and Kelly, 2020). Classic population genetic theory based on models of hard selection predicts rapid coevolution between selfing rate and inbreeding depression; higher selfing rates produce an overabundance of homozygous individuals, thereby purging populations of (at least partially) recessive deleterious mutations more efficiently (Lande and Schemske, 1985). Despite this prediction, many species have intermediate selfing rates (i.e., are “mixed mating”) with a severity of inbreeding depression as great as that found in outcrossing populations (Goodwillie et al., 2005; Winn et al., 2011). More generally, evidence for purging in wild populations is equivocal (Byers and Waller, 1999; Keller and Waller, 2002). Various explanations for these observations include alternative genetic architectures causing inbreeding depression (Gervais et al., 2014), selective interference among loci (Winn et al., 2011), and the impacts of population bottlenecks or similar disequilibrium scenarios (Spigler et al., 2017; Waller, 2021). However, the mutation load that gives rise to inbreeding depression could also be maintained in part by soft selection on deleterious alleles in subdivided populations where genotypes are clustered (Charlesworth and Charlesworth, 1987; Theodorou and Couvet, 2006; Cheptou and Schoen, 2007; Ho and Agrawal, 2012).

Consider the scenario where selfed and outcrossed individuals vary in their ability to acquire resources and fitness hinges on competitive ability. A selfed individual is expected to perform poorly when competing against superior outcrossed competitors. However, a selfed individual competing exclusively against other selfed individuals has the potential to achieve high absolute fitness. This fitness level may even rival that of an outcrossed individual forced to compete against other outcrossed competitors. These dynamics lead to seemingly counterintuitive evolutionary and ecological outcomes because of the contrasting impacts on individual vs. mean fitness: under soft selection: individual fitness will depend on the genetic composition of the population, sheltering selfed individuals from selection and maintaining mutation load in highly selfing populations, yet mean fitness can be the same among populations of contrasting load (Agrawal, 2010; Agrawal and Whitlock, 2012). Even the increased variance and greater opportunity for selection within mixed-mating populations need not affect population growth if fitness gains of outcrossed individuals and losses of selfed individuals are a zero-sum game (Holsinger and Pacala, 1990; Agrawal, 2010; Abu Awad et al., 2014). It is worth noting that although the “local” mean trait and fitness values in soft selection are often considered at the level of population or deme, the spatial scale at which relevant ecological interactions determine fitness, i.e. the ecological neighborhood (sensu Addicott et al., 1987), can be smaller. In this way, mutation load could also be maintained in mixed-mating populations when habitat heterogeneity leads to clusters of individuals that vary in frequency of selfed and outcrossed individuals. The conditions for soft selection on deleterious alleles contributing to inbreeding depression in competitive ability should be common in plant populations, where intraspecific competition is a ‘primary interaction’ (Harper, 1977; Weiner, 1985; Silvertown and Charlesworth, 2001) and limited seed dispersal distance often results in fine scale spatial genetic structure. This structure manifests as the clustering of genetically related individuals, especially noticeable during the seedling and juvenile stages, where high densities prevail (Loveless and Hamrick, 1984).

However, the extent to which individual fitness depends on the composition of competitors, i.e., the ‘softness’ of selection, and/or selection strength should change with density (Agrawal, 2010; Laffafian et al., 2010; Agrawal and Whitlock, 2012; Ho and Agrawal, 2012; Yun and Agrawal, 2014; Bell et al., 2021). In addition to the rate of resource capture, plant growth depends on how efficiently acquired resources are transported and the maximum capacity for resource absorption. Assuming a fixed amount of resources, low densities correspond to high per capita resource availability. In this scenario, fitness will be constrained by a genotype’s maximum capacity for resource intake, which is subject to hard selection. As per capita resource availability declines, maximum size is less attainable by any genotype, and individual fitness will depend more on resource capture relative to competitors. In the case of inbreeding depression, outcrossed individuals can gain a further advantage preempting resources under these conditions if they germinate earlier than selfed seeds (Husband and Schemske, 1996) and/or have larger initial seedling sizes (Cheptou and Schoen, 2003; Sandner et al., 2021). Combined with faster rates of resource capture, outcrossed plants can dominate and suppress selfed conspecifics (Schmitt and Ehrhardt, 1990). The asymmetry in competition between outcross and selfed individuals should result in exaggerated fitness differences, i.e., inbreeding depression, in mixed neighborhoods as density increases, unless there are concomitant increases in mortality. This should manifest as greater inequalities in fitness components such as plant size and/or reproduction (Thomas and Weiner, 1989; Weiner, 1990) in neighborhoods where outcrossed and selfed plants compete compared to homogeneous neighborhoods composed solely of either selfed or outcrossed plants. A limited number of experimental plant studies have revealed patterns of individual plant performance consistent with the outcome of asymmetric competition between selfed and outcrossed plants (Schmitt and Ehrhardt, 1990; Damgaard and Loeschcke, 1994; Koelewijn, 2004) and at least one study in *Drosophila* demonstrated a clear link between the strength of inbreeding depression and density-dependence (Yun and Agrawal, 2014). A corollary of a shift in softness of selection with density is that the strength of density-dependence itself can differ between solely outcrossed or solely selfed neighborhoods. Outcrossed individuals may begin to interfere with each other at a lower density than selfed plants and experience greater density dependence. However, stronger density-dependence could occur in selfing neighborhoods if selfed individuals have lower tolerance for stress associated with competition (Fox and Reed, 2011; Sandner et al., 2021).

Previous work assessing at least one axis of soft selection (i.e., density or frequency-dependent, see Wallace, 1975) on inbreeding depression in plants have used an individual-level experimental approach, typically by surrounding focal individuals with different frequencies and/or densities of selfed or outcrossed competitors. Such experiments have demonstrated how performance of selfed individuals may decline with increasing frequency of outcrossed individuals, but the influence of density on inbreeding depression in terms of both its presence and direction of effect remains uncertain (Waller, 1985; Schmitt and Ehrhardt, 1990; Wolfe, 1993; Cheptou et al., 2001; Cheptou and Schoen, 2003; Koelewijn, 2004; Lhamo et al., 2006). The extent to which expected density patterns manifest at the neighborhood or population level remains a less explored aspect. A complementary approach is to create purely selfed, purely outcrossed, or mixed competitive neighborhoods wherein seedlings recruit unconstrained and resultant densities span a gradient mimicking densities in the field. In addition, mortality becomes part of the population dynamics rather than an event to result in plant replacement. Although it is possible to examine both individual- and population-level dynamics in some organisms, it is difficult to do for plants at high densities.

We tested population-level predictions of soft selection (Bell et al., 2021) affecting inbreeding depression in the mixed-mating biennial *Sabatia angularis* (Gentianaceae) by creating experimental competitive neighborhoods comprised of completely selfed, completely outcrossed, or mixed individuals in pots across a density gradient. We focused on the juvenile stage and followed plants from germination through juvenile survival and growth. We tested the effect of genetic composition on mean juvenile size and the extent to which this effect changes with density. If there is soft selection on competitive ability, then mean plant size will be independent of the genetic composition where competition for resources occurs. Then, we tested whether size inequalities among plants within competitive neighborhoods depended on genetic composition and whether inequality increases with plant density. If competition is asymmetric between selfed and outcrossed plants, mixed-mating neighborhoods should exhibit stronger size hierarchies than homogenous neighborhoods composed solely of either selfed or outcrossed plants. Moreover, the degree of inequality (and thus selection strength) should increase with density, unless there is concomitant density-dependent mortality. To complement these tests, we also considered the effects of genetic composition on germination rate, germination timing, and survival.

## 2 Methods

### 2.1 Study species

*Sabatia angularis* is a biennial native to grasslands and disturbed sites in eastern North America. Seeds germinate throughout late spring and summer and develop into rosettes that continue to grow until the onset of cool conditions in October. Rosettes overwinter before bolting and flowering July-August. *S. angularis* is self-compatible, and populations range from mixed mating to highly outcrossing, with a large proportion of the variance in mean outcrossing rate explained by variation in population size (Spigler et al., 2010). Inbreeding depression occurs across the lifecycle, and its cumulative impact on fitness can be substantial (Dudash, 1990) but differs across populations indicating differences in population mutation load (Spigler et al., 2017).

Plant density in wild *S. angularis* populations is highly variable. Primary seed dispersal is passive; the miniscule seeds (approximately 300μm in diameter) fall by gravity from dry dehiscent capsules. Consequently, most juveniles are found within 1m of the adult plant (Dudash, 1991). With each plant producing an average of ~30 fruits containing ~750 seeds per fruit, juvenile densities can be very high, even accounting for low germination rates (<1-3% in wild populations; Walker, 2023). Juvenile density can reach up to the equivalent of 82 juveniles per 100cm^2^ (Walker, 2023). Prior work in wild *S. angularis* populations established that juvenile size is density-dependent at the scale of centimeters (Walker, 2023).

### 2.2 Experimental design

Plants were grown in pots, with each pot representing an ecological neighborhood; therefore, we refer to pots throughout as neighborhoods. Seeds used to populate neighborhoods represent paired sets of outcrossed and selfed seed from 21 maternal plants (i.e., families). These seeds originated from pollinations conducted in Spigler (2017). In brief, Spigler (2017) collected seeds from a wild population, raised maternal plants under controlled, pollinator-free conditions, and hand-pollinated paired sets of flowers on each plant with self and outcross pollen. Outcross pollen represented a mixture of pollen collected from five unrelated individuals. Maternal plants were likely highly heterozygous, as the originating population was large (>1000 adults; “UB5” in Spigler, 2018), and such large populations are highly or near completely outcrossed (Spigler et al., 2010).

Our goal was to create local neighborhoods representing a gradient of densities across each of three mating system treatments (“neighborhood composition”): 100% selfed, 100% outcrossed, and “mixed” (50/50% selfed/outcrossed seed). To achieve this, we planted a series of seed densities (10, 20, 40, 80, and 160; 320 and 640 seeds when seed quantities allowed), hereafter “planting densities’’, for each family-cross combination in 60mm x 60mm x 40mm (L x W x D) pots filled with a 3:1 ratio of Pro-Mix BX (Premier Horticulture) and Turface All Sport (Profile Products). Although all seeds within a pot originate from a single maternal family, genetic relatedness to neighbors will vary across treatments. This variation extends to homozygosity, which is intentional as part of the design. Our goal is to test for soft selection on exposed deleterious recessive alleles related to competitive ability. To mimic seed rain in natural populations, we combined outcrossed and selfed seeds for mixed-mating competitive arenas prior to planting; accordingly, we could not identify individual plants as either selfed or outcrossed. We planted additional replicates for planting densities <80 to account for low germination rates in the species. We were unable to create the full factorial (planting density x cross type) for some families due to low seed set. A full table of families and sample sizes per planting density treatment can be found in the **Supplementary Information** (**Supplementary Table S1**). In total, we started with 429 pots (N=159 selfed, N=154 outcrossed, and N=116 mixed) across the 21 families. Pots were randomly arranged into trays (32 pots/tray) and placed in a controlled growth chamber kept at 25-30°C/15°C day/night with 14 daylight hours. Trays were regularly watered and rotated within the chamber. Approximately 3mo after planting, half of the pots showed no germination (no difference across neighborhood composition treatments, *χ* ^2 =^ 2.4, p=0.31); we therefore applied 50 ppm of gibberellic acid to help break physiological dormancy, found to be effective for *S. angularis* in other studies (Wennerberg, 2005). Across the study, germination occurred in 236 pots (N=77 selfed, N=88 outcrossed, and N=71 mixed), 44 of which were treated with GA, with no difference among neighborhood types (*χ* ^2 =^ 2.58, *P*=0.28). We note that we use the term “juveniles” to include seedlings and rosettes, given a neighborhood can have a mixture of new seedlings and seedlings that have grown into rosettes at any given point.

We counted juveniles per competitive neighborhood weekly (“juvenile density” or “density”). Changes in density across time can be a function of mortality and new germination. To calculate germination and mortality, we made two simplifying assumptions. First, the maximum number of juveniles per pot found at any census represents the total number of juveniles per pot. Second, any decrease in population size from the maximum to the final census is due to mortality. We also determined the median date of germination per pot as the sampling date at which ≥50% of the maximum number of plants were found.

We took measurements at two time points relative to the overall average date that germination began across pots (“mean onset of germination”). These two time points were ~30d and 90d after mean onset of germination (see **Supplementary Figure S1** for example photos). Leaf surface area (mm^2^) of seedlings was calculated as the length from tip to tip of the opposing cotyledons multiplied by cotyledon width. Seedlings develop into rosettes composed of opposite and decussate pairs of flattened leaves, forming a cross that can be interpreted as axes of an ellipse. The lengths of the major and minor axes were measured and the product of the two measurements used to estimate rosette leaf area (mm^2^). At 30d, 149 pots had at least one juvenile (see **Supplementary Table S1**). We measured leaf area for 90% of juveniles (1648/1824) across all 149 pots (“initial size”). By 90d, 184 pots had at least one juvenile; we measured all 1365 plants across pots (“final size”). Measurements were made by hand or based on photographs using ImageJ software (Schneider et al., 2012). We used size as an indicator of competitive ability, or at least its outcome. Though we recognize that size can vary due to timing of germination, initial (seedling) size, and/or biomass conversion efficiency, larger size in competitive environments often indicates superior resource acquisition and therefore competitive success (Weiner, 1990). Leaf area is also easily quantified across thousands of plants.

To address the extent to which competition may be symmetric or asymmetric, we used measurements from each pot to calculate a Gini coefficient (G) for each of the two time points. The Gini coefficient is often used in plant studies to quantify size hierarchies (Weiner and Solbrig, 1984; Damgaard and Weiner, 2000). It is calculated as the area between the line approximating the cumulative ranking of all plants per in a competitive neighborhood, known as the Lorenz curve, and the line of equality, which represents the relationship that would occur if all individuals were the same size. G ranges from 0 to 1, with 0 denoting perfectly equally sized individuals and 1 denoting the highest possible inequality (whereby one individual possesses all biomass). G should increase as competition becomes more asymmetric. We calculated G for pots with at least 5 juveniles using the R package ‘*ineq’* (Zeileis and Kleiber, 2014).

We included the two time periods because of contrasting expectations. Early in growth, juveniles are less likely to compete due to their small above and below-ground sizes (Schwinning and Weiner, 1998). Therefore density is not expected to impact plant size, our metric of growth, for any cross type. However, we may expect to see initial size differences across neighborhood types (i.e., a main effect of neighborhood composition) if outcrossed plants germinate earlier or have larger initial sizes. This could also lead to greater size inequalities in mixed neighborhoods (estimated by G), but we would not expect density to affect this inequality. After a period of growth such that plants have expanded their zone of influence (e.g., Weiner and Damgaard, 2006) and engage in competition for light and/or soil resources, we expect to see effects of density on size. Moreover, if there is competitive asymmetry between selfed and outcrossed plants, we expect to see its signatures at our final time point as differences in Gini coefficient across neighborhood types and an increase in inequality with density (Weiner and Thomas, 1986).

### 2.3 Statistical analyses

#### 2.3.1 Germination and survival

We tested for differences in germination across pots in two steps as a hurdle model. First, we treated germination as a binary variable; pots either had at least one juvenile or none. We tested the influence of neighborhood composition (categorical: mixed, selfed, or outcrossed), planting density (continuous), and their interaction on the probability of at least one germinant using a generalized linear mixed model proc glimmix in SAS 9.4 (Copyright ^©^ 2012-2020, SAS Institute Inc., Cary, NC, USA). Planting density and its interaction with neighborhood were included, as higher seed numbers increase the likelihood of germination, a probability that may vary with neighborhood type. We initially included maternal family, tray, and gibberellic acid (“GA”) treatment (yes/no) as random effects and a random group term that can account for any heterogeneity of variances among neighborhood composition treatments and improved model fit. We treated GA as random because we have no interest in examining or measuring the effects of that treatment; rather this acknowledges their variability and treats them as blocks (GA treated pots started germination and growth later). However, in no case (this model or following models) was GA significant according to a Wald test, therefore we proceeded only with family and tray effects. Second, for pots containing at least 1 juvenile, we tested whether neighborhood composition influenced germination rate (i.e., percent germination). We used a weighted mixed model regression to test the effects of neighborhood composition on the arcsin square root of germination percentage (proc mixed). Germination rate could change with seed density if site limitation occurs. Therefore, we also included planting density and its interaction with neighborhood composition. We included the random effects of tray and family and used planting density as the weighting factor.

Differences in plant size among neighborhoods could arise without inbreeding depression for competitive ability or growth if selfed plants germinate significantly later than outcrossed ones. Therefore, we tested for differences in median germination date among neighborhoods using a general linear mixed model (proc mixed). (For pots with a single juvenile, the date of germination was used as the response.) We included neighborhood composition (categorical) and planting density (continuous) as main effects, tray and family as random effects, and a random group term to account for significant heterogeneity of variances among neighborhood composition treatments.

We employed a hurdle model to evaluate the effect of neighborhood competition on mortality and to determine whether mortality was density dependent. The distribution of mortality was irregular (37% pots with no mortality). Therefore, we first used a generalized linear mixed model (proc glimmix) to model the probability of mortality occurring (0 vs 1) and then used a weighted general linear mixed model (proc mixed) to model mortality rate (i.e., the proportion of juveniles that died) in pots where mortality occurred. We highlight again that after germination has occurred, we focus on the effects of juvenile density vs planting density. We calculated the mortality rate for each pot by subtracting the number of juveniles at the final census from the peak number of juveniles seen during the study and then dividing by that peak number. We used an arcsine square root transformation on mortality rate. In both binary and linear regression models, we included neighborhood composition, peak density (continuous, log_10_-transformed), and their interaction as fixed effects. We also included family and family by neighborhood composition interaction as random effects and accounted for heterogeneity of variances among neighborhood composition types as above. In our model of mortality rate, we incorporated peak density (untransformed) as a weighting factor and included the date of peak density as a covariate, given mortality typically increases over time.

For these and all following analyses, we tested model assumptions (normally distributed residuals, no significant heteroscedasticity among groups) and assessed presence of highly influential data points based on restricted likelihood distance, Cook’s Distance, covariance ratios, and/or the absolute value of studentized residuals. We removed data points identified as influential or outliers when they qualitatively changed the outcome (results including all data points are provided in **Supplementary Information**). We removed covariates when p>0.05 to maximize statistical power. When the main effect of neighborhood composition was significant, we tested all pairwise comparisons with contrast statements. When the interaction between neighborhood composition and density was significant, we conducted *post hoc* statistical analyses to examine: (1) whether the effect of density was significant for all treatments (i.e., if slopes were significantly different from zero); (2) pairwise differences in slope estimates across different neighborhood compositions; and (3) differences in the response variable across neighborhoods at specific density levels. We present marginal means and 95% confidence intervals (CI) for the estimates in the results. Where necessary, these values have been back-transformed to their original scale for clearer interpretation.

#### 2.3.2 Plant size

We evaluated density-dependence by examining the effect of observed juvenile density per pot on leaf surface area. Although there are several ways that density-dependence can be evaluated, this is a common approach in plant studies (e.g., Ramula and Buckley, 2009). We used a general linear mixed effects model to evaluate the effects of neighborhood composition, juvenile density as observed on the associated census date (log_10_-transformed), and their interaction on leaf surface area (log_10_-transformed) at each time point (initial, final), separately (proc mixed). Because both leaf area (response) and density (predictor) were log_10_-transformed, the slope can be interpreted as the percent change in area given a 1% change density; thus a significant interaction between neighborhood composition and density indicates a disproportionate impact of density on at least one of the neighborhood types. We treated individual juvenile measurements per pot as repeated measures to account for non-independence among plants within a pot and included maternal family and family*neighborhood composition as random effects. In addition, we accounted for significant heterogeneity of variances among neighborhood composition treatments. Since variation in plant size can be affected by differences in germination timing as well as competitive ability, we included median germination date of each pot (or date of germination for pots with a single juvenile) as a covariate.

If selfed and outcrossed individuals differ in competitive ability, such that outcrossed individuals dominate and suppress selfed ones, there should be greater size inequalities in mixed neighborhoods, estimated as G, compared to neighborhoods composed of either 100% selfed or 100% outcrossed individuals (“homogeneous”). Asymmetric competition should also result in greater inequalities at greater densities. We used general linear mixed models to test whether G varied as a function of density, neighborhood type (mixed vs. homogeneous), and their interaction, accounting for a random family and family by neighborhood interaction effects (proc mixed). Median germination date was again included as a covariate to account for potential differences in germination timing, as size inequalities may be influenced by the age of juveniles (Arenas and Fernández, 2000). However, although this covariate was significant, its inclusion (i) caused several pots with extremely late median germination dates (~30d past the next latest) to be highly influential and (ii) either had no effect on or worsened model fit (delta AIC>2). Therefore, we removed it. We also conducted a complementary analysis to test for signatures of asymmetric competition, recognizing that categorizing selfed and outcrossed neighborhoods into a single “homogeneous” category may be overly simplistic. We converted neighborhood composition from categorical to the frequency of selfed seeds based on planting density (outcrossed=0, mixed=0.5, selfed=1), like the approach used by Weis et al. (2015). We tested for a quadratic relationship between G and density, including a linear and quadratic selfed frequency term (continuous), along with an effect of density (log_10_-transformed) and its interaction with selfed frequency. We included the same random effects as in the categorical analysis. Plots showing the results of our size analyses were created using the ‘ggplot2’ package in R (Wickham, 2016).

## 3 Results

Results from the binary regression indicated that the expected proportion of pots where germination occurred was lower for selfed neighborhood (0.34, 95%CI: 0.21-0.51) compared to mixed (0.50, 95%CI: 0.33-0.67) and outcrossed 0.44 (95%CI: 0.28-0.61) neighborhoods, but differences were not statistically significant (**Table 1**). This proportion increased significantly with planting density, and there was no significant interaction between density and neighborhood composition (**Table 1**). Germination rate in pots with at least one juvenile was the same across selfed (0.06, 95%CI: 0.04-0.08), mixed (0.06, 95%CI: 0.04-0.08, and outcrossed (0.08, 95%CI: 0.05-0.10) neighborhoods (**Table 1**). Planting density had a significantly negative impact on germination rate, suggesting site limitation (**Table 1**). The interaction between density and neighborhood composition was not significant. However, reintroducing a single influential data point alters the results, revealing a significant slope only in mixed neighborhoods (**Supplementary Table S2**). Median germination date did not differ across neighborhood composition types (*F*_2,177 =_ 0.64, *P*=0.53) nor was it affected by planting density (*F*_1,177_ = 0, *P* = 0.97). However, variances differed among neighborhood types (*χ* ^2 =^ 6. 7, *P*=0.04), with the greatest and least variance in median germination date among outcrossed and selfed neighborhoods, respectively.

**Table 1:** Model results testing the effect of neighborhood composition and planting (seed) density on germination.

	Probability of germination (0 vs 1)				Germination rate			
Fixed effects	Num df	Den df	*F*	*P*	Num df	Den df	*F*	*P*
Neighborhood composition	2	347	2.00	0.14	2	174	0.63	0.53
Planting density	1	347	26.7	<0.0001	1	174	6.98	0.009
Neighborhood × Density	2	347	0.72	0.49	2	174	1.74	0.18
Random effects			*Z*	*P*			*Z*	*P*
Tray			3.52	0.0002			1.32	0.09
Family			1.04	0.15			0.59	0.28

Neither the probability of mortality nor the mortality rate (in neighborhoods where at least one juvenile died) were affected by neighborhood composition or its interaction with density (**Table 2**). However, both measures of mortality were density-dependent (**Table 2**). Mortality occurred in 62.6% of neighborhoods, and marginal mean mortality rates were 0.60 (95%CI: 0.47-0.72), 0.54 (95%CI: 0.40-0.68), and 0.59 (95%CI: 0.47-0.71) for selfed, mixed, and outcrossed neighborhoods, respectively. As expected, mortality rate was significantly greater when density peaked earlier (**Table 2**).

**Table 2:** Model results testing the effect of neighborhood composition, density, and their interaction on mortality.

	Probability of mortality (0 vs 1)				Mortality rate			
Fixed effects	Num df	Den df	*F*	*P*	Num df	Den df	*F*	*P*
Neighborhood composition	2	36	0.02	0.98	2	32	0.79	0.46
Peak density	1	160	41.37	<.0001	1	80	5.92	0.02
Neighborhood × Density	2	160	0.34	0.71	2	80	1.63	0.20
Date of peak density	–	–	–	–	1	80	14.2	0.0003
Random effects			*Z*	*P*			*Z*	*P*
Family			.	.			1.34	0.09
Family × Neighborhood			1.33	0.09			1.4	0.08

**Figure 1:**
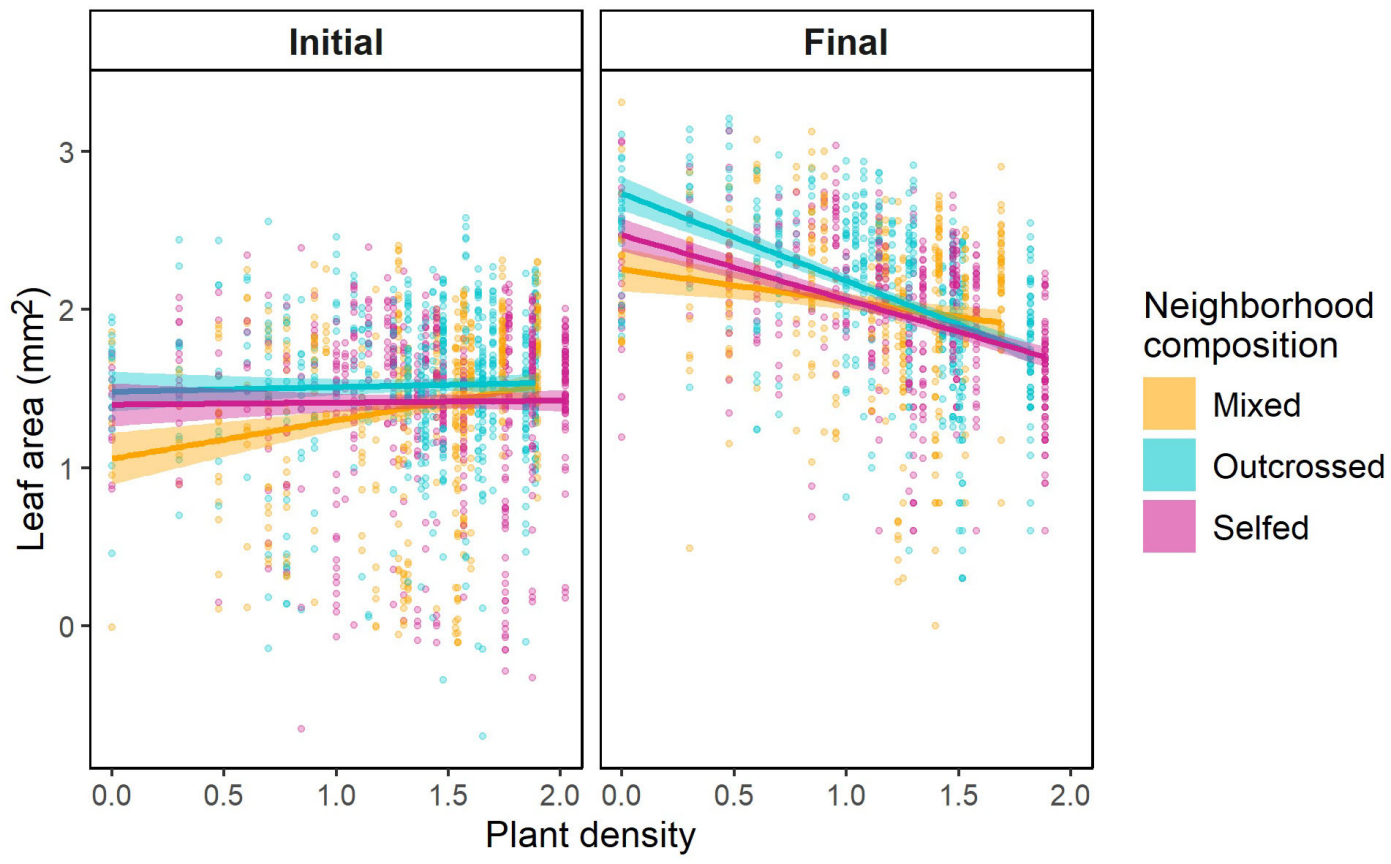
Scatterplots and prediction lines for changes in juvenile leaf area with density in pots composed of selfed seed (pink), outcrossed seed (blue), or a mixture (orange). Results are shown for the **(A)** initial census and **(B)** final census. Dots represent individual plant measurements; regression lines and 95%CI envelopes are based on general linear models considering covariates, random effects, and treating individual measurements as repeated measures within plots (see Methods). Plant density and leaf area are log_10_-transformed.

At the initial census, plants were relatively small across all densities (**Figure 1**; **Supplementary Figure S1**). Marginal mean leaf area (95%CI) for selfed, mixed, and outcrossed neighborhoods was 26.8mm^2^ (21.9-33.0), 27.3mm^2^ (21.5-34.7), and 34.33mm^2^ (28.2-41.9), respectively. The interaction between neighborhood composition and juvenile density on size was significant (**Table 3**). Whereas plant size did not vary with density in selfed or outcrossed neighborhoods, the slope was significantly positive in mixed neighborhoods (**Figure 1**; **Supplementary Table S3**). However, we only detected a significant difference in slopes between mixed and selfed neighborhoods (**Supplementary Table S3**). Probing the interaction further, *post-hoc* comparisons at different densities revealed that average plant size was smaller in mixed neighborhoods compared to either selfed or outcrossed neighborhoods at below-average densities, but not at higher densities (**Figure 1**; **Supplementary Table S4**). Median germination date strongly affected juvenile size, with earlier germination leading to larger plants (**Table 3**). The random interaction between family and neighborhood composition was significant, suggesting the effect of the latter varies across families. Size inequality based on the Gini coefficient tended to be greater in mixed neighborhoods compared to homogeneous ones at the initial census (0.43 ± 0.02SE vs. 0.38 ± 0.02SE, respectively), but this difference was not significant (**Table 4**). Neither density nor its interaction with composition affected inequality at this stage (**Table 4**). Results were similar when we considered the frequency of selfed seeds based on planting density, with no significant linear (*F*_1,55 =_ 2.94, *P*=0.09) or quadratic (*F*_1,55 =_ 3.7, *P*=0.06) frequency effects or effect of density (*F*_1,55 =_ 1.07, *P*=0.31).

**Table 3:** Model results testing the effect of neighborhood composition, density, and their interaction on plant size at initial and final censuses.

	Initial census				Final census			
Fixed effects	Num df	Den df	*F*	*P*	Num df	Den df	*F*	*P*
Neighborhood composition	2	34	5.28	0.01	2	36	13.2	<0.0001
Density	1	1583	6.16	0.01	1	1302	155.4	<0.0001
Neighborhood × density	2	1583	4.4	0.01	2	1302	11.2	<0.0001
Median germination date	1	1583	70.12	<0.0001	1	1302	30.9	<0.0001
Random effects			*Z*	*P*			*Z*	*P*
Family			0.17	0.43			1.3	0.10
Family × Neighborhood			2.5	0.006			2.5	0.01

**Table 4:** Results of mixed models testing the effects of neighborhood type and density on size inequality, estimated as the Gini coefficient.

	Initial census				Final census			
Fixed effects	Num df	Den df	*F*	*P*	Num df	Den df	*F*	*P*
Neighborhood type (mixed vs. homogeneous)	1	56	1.01	0.32	1	48	2.19	0.15
Density	1	56	2.09	0.15	1	48	9.66	0.003
Neighborhood × Density	1	56	0.31	0.58	1	48	0.68	0.41
Random effects			*Z*	*P*			*Z*	*P*
Family			.	.			0.78	0.22
Pairwise contrasts		df	*t*	*P*		df	*t*	*P*
Mixed vs. homogeneous		56	1.57	0.12		48	2.86	0.006

By the final census, plant size increased 4-fold (**Supplementary Figure S1**). Mean plant size per neighborhood decreased significantly with density across all neighborhood composition treatments (**Figure 1**; **Table 3**; **Supplementary Table S5**). However, the interaction between density and neighborhood composition was significant (**Table 3**). Density-dependence was significantly greater in outcrossed neighborhoods compared to selfed or mixed neighborhoods (**Supplementary Table S5**). In fact, the slope of the log-log relationship between size and density for outcrossed neighborhoods (*β*=-0.60 ± 0.05SE) was 1.7-2.4x greater than the slope for the other two neighborhoods (**Supplementary Table S5**). The difference in size between outcrossed neighborhoods and either mixed or selfed ones was large and significant at low densities (**Supplementary Table S6**) but declined with density and ultimately converged across neighborhood treatments (**Figure 1**). In fact, mean size was statistically similar across treatments at densities of 10 juveniles and greater (log_10_ density = 1; **Supplementary Table S6**). Plant size was also smaller in pots with later median germination dates (**Table 3**). The interaction between family and neighborhood composition was significant (**Table 3**). Variation in size among pots was significantly greater among mixed neighborhoods (0.23 ± 0.02SE) than among either outcrossed (0.17 ± 0.01SE) or selfed (0.15 ± 0.01SE) neighborhoods (*χ* ^2 =^ 23.05, P<0.0001).

**Figure 2:**
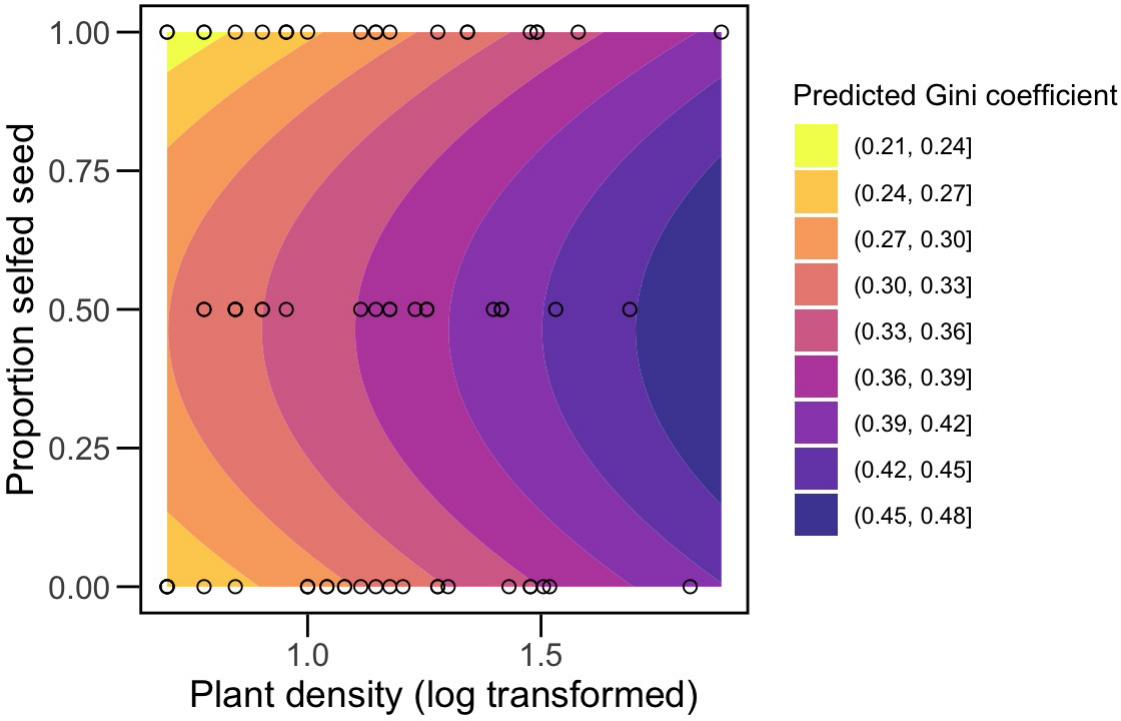
Contour plot illustrating the predicted effects of density (x-axis, log_10_-transformed) and frequency of selfed seed planted per pot (y-axis) on size inequality, estimated as the Gini coefficient, at the final census. Shading of contours illustrates a gradient from lower G values to higher values as indicated in the legend. Dots show the distribution of data points along the density gradient for each of the three neighborhood compositions, plotted as frequencies. G increases with density. For a given density, the contour shows that inequality is predicted to be greater at intermediate frequencies. We emphasize that mixed neighborhoods may have deviated from 0.5 after germination and mortality.

Size inequalities were greater within mixed neighborhoods (mean *G* = 0.36 ± 0.02SE) compared to homogeneous neighborhoods (mean *G* = 0.30 ± 0.01SE) at the final census. The main neighborhood effect was initially masked by the non-significant interaction term. However, it became evident through the highly significant pairwise contrast between neighborhood types (**Table 4**). A significant main effect of neighborhood type is similarly detected when the interaction is removed, even when 2 outliers are included (**Supplementary Table S7**). Size inequality also increased significantly with density (**Table 4**). We found similar results when modeling neighborhood composition as the frequency of selfed seed. The linear (*F*_1,47 =_ 6.63, *P*=0.01) and quadratic (*F*_1,47 =_ 8.4, *P*=0.006) terms for frequency were significant, indicating inequality was greatest in 50% selfed populations (**Figure 2**). *G* also increased with density (*F*_1,47 =_ 11.62, *P*=0.001). As in the categorical analysis, the interaction term was not significant (*F*_1,47 =_ 1.05, *P*=0.31).

## 4 Discussion

Our findings are consistent with differences in competitive abilities of outcrossed and selfed *S. angularis* plants and illustrate how the strength of density dependence and selection can vary with the genetic composition of an ecological neighborhood. We demonstrated equivalent mean leaf area, a metric of plant growth, across selfed, outcrossed, and mixed neighborhoods at high densities under competition, consistent with population-level expectations of soft selection (Whitlock, 2002; Agrawal, 2010; Bell et al., 2021) and the constant final yield rule (Weiner and Freckleton, 2010). However, at low densities, we see a pronounced contrast in mean plant size between selfed and outcrossed neighborhoods, implying a gradient from hard to soft selection. We further detected key signs of asymmetric competition (Weiner, 1990): greater size inequality in mixed neighborhoods and an increase in size inequality with density. These patterns became apparent only during the final census, after sufficient opportunity for competition to unfold.

We prioritized competition during the juvenile stage to mimic the growing season before the onset of overwintering in *S. angularis*, considering the known relevance of density-dependence at this stage in wild populations (Walker, 2023). Although this represents only part of the life cycle, population growth of many herbs is sensitive to juvenile growth and survival (Cook, 1979; Schmidt and Lawlor, 1983; Horvitz and Schemske, 1995). Our prior work in common garden experiments and wild populations substantiates juvenile size as a relevant component of fitness in *S. angularis* that influences survival to flower (Spigler et al., 2017; Walker, 2023) and flower number (based on data from Spigler et al., 2017). Moreover, we also prioritized capturing patch- or population-level dynamics. Studies tracking individual plants are important as they allow for estimates of individual fitness but require arranging plants within simple, uniform distributions (e.g., Waller, 1985; Cheptou and Schoen, 2003; Lhamo et al., 2006; Koelewijn, 2004). In wild populations, however, spatial heterogeneity combined with limited dispersal leads to clustering and, particularly for herbaceous plants, fine-scale spatial interactions occurring on the order of centimeters (e.g., Purves and Law, 2002). Tracking individuals in *S. angularis* would logistically require transplanting a selection of small rosettes and could fail to capture early, fine-scale ecologically relevant dynamics. We interpret the dynamics shown here and explore their potential implications for natural populations, including how soft selection might mitigate the genetic load associated with inbreeding depression and its demographic consequences.

### 4.1 Population-level signatures of soft selection against selfed individuals in *S. angularis*

It seems intuitive, though not necessarily true that the softness of selection should increase with the intensity of competition, at least for traits that affect growth (Laffafian et al., 2010). Without competition for resources there is no race to acquire resources, and, where resources are limited, there is less opportunity for individuals to reach their maximal size. Thus, plant growth at low densities should depend solely on their efficiency in converting resources into biomass (Agrawal, 2010; Weiner and Freckleton, 2010). As per capita resource availability declines, emphasis shifts to resource consumption. This provides an opportunity for certain genotypes to capture a greater share of resources, thereby suppressing the growth of less competitive individuals (Weiner and Freckleton, 2010). These dynamics should lead to two distinctive signatures, which we demonstrate here: an interaction between density and genetic composition and greater fitness variation in neighborhoods with a heterogeneous genetic composition compared to those with a homogeneous composition. Weis et al. (2015) found similar population-level patterns in their study investigating hard and soft selection on seedling emergence time at two densities in *Brassica rapa.* Their findings further demonstrated that the strength of soft selection was five times greater than that of hard selection in the high-density treatment. Though we could not quantify selection strengths, our results clearly illustrate a substantial impact of neighborhood composition on mean size at low densities that diminishes and ultimately disappears at high densities. This pattern occurs because outcrossed plants, while intrinsically larger in the absence of competition, experience stronger density dependence in outcrossed neighborhoods, which also provides evidence of their superior competitive ability. Interestingly, by the final census, mixed neighborhoods exhibited a density response similar to that of selfed neighborhoods. This could be linked to the difference in mean size that we saw at the initial census at low densities. We suspect this initial difference could be a product of stochasticity. We sowed ‘mixed’ treatments with an equal mix of selfed and outcrossed seeds, but low and variable germination rates likely caused the actual composition of mixed neighborhoods to deviate from parity, especially at low densities. Ultimately, if we assume that plant size is related to fitness, our results suggest that, when densities are low, outcrossed demes will contribute more to the next generation than mixed or selfed demes, consistent with hard selection. In contrast, all three types would contribute equally to the next generation at high densities, even though selfed demes harbor more genetic load, consistent with soft selection.

We also found evidence that mixed neighborhoods exhibited greater size inequality at the final census, an expected pattern if competition between outcrossed and selfed individuals is asymmetric. This result was also evident when we analyzed neighborhood type in terms of the frequency of selfed seed. In our study, competition could occur for soil resources and, at higher densities, light and space as rosettes start to overlap (see **Supplementary Figure S1**). Competition for light is the most likely mechanism of asymmetric resource competition in plants, as a larger plant shading a smaller plant creates a winner-takes-all scenario for light availability (Weiner, 1990). In contrast, root competition has traditionally been viewed as symmetric, though this assumption may not hold true, especially in low-nutrient soil conditions (Brown et al., 2019; Rasmussen et al., 2019). Outcrossed individuals in mixed neighborhoods could gain an advantage at resource capture if they germinate first, have larger initial sizes, or more quickly acquire and/or convert resources. We did not see significant differences in median germination date among neighborhoods, and mean size was similar between at least outcrossed and selfed neighborhoods at the initial census, pointing toward resource acquisition. It is possible that at least some of the greater inequality in mixed neighborhoods at the final census is an artifact of simply mixing larger outcrossed and smaller selfed plants rather than from asymmetric competition. Although we cannot not entirely dismiss this possibility, we also found that inequality increased with density, another signature of asymmetric competition (Weiner and Thomas, 1986; Weiner, 1990). The increase in inequality with density in mixed neighborhoods was not steeper than in homogeneous ones, as we predicted, suggesting some level of asymmetric competition among genotypes in all neighborhood types. Still, mixed neighborhoods consistently displayed larger size hierarchies, even as average size declined and converged across all neighborhood types with increasing density. This indicates that greater size inequality in mixed neighborhoods cannot solely be attributed to combining differently sized plants. It is important to note that an increase in inequality with density need not be the case. For example, Waller (1985) found no consistent effect of density at a single point in time and, when comparing across time periods, observed the greatest increase in inequality at low density, emphasizing the role of intrinsic differences between selfed and outcrossed individuals. In contrast, if we compare G across our two time periods, we see a decrease in inequality for low density neighborhoods, suggesting the effects of intrinsic differences decrease over time, while inequality increased at high densities (data not shown). Asymmetric competition in our neighborhoods could also contribute to explaining similar trend lines between mixed and selfed neighborhoods at the final census, e.g., if selfed individuals incur disproportionate losses when competing with outcrossed individuals but outcrossed individuals do not reap disproportionate gains.

Our results could alternately be viewed through the lens of kin competition. Some research indicates that intraspecific competition in plants declines with the relatedness of competing individuals (Dudley and File, 2007; Ehlers and Bilde, 2019). For example, Dudley and File (2007) found that related individuals under competition reallocated biomass away from competition-related traits, such as lateral root growth. Since plants within each competitive neighborhood in our study shared the same mother, those within selfed neighborhoods were more closely related than those within outcrossed (or mixed) neighborhoods. Selfed neighborhoods never outperformed outcrossed neighborhoods in terms of plant size, contrary to expectations of kin cooperation, although one could interpret lower density-dependence in selfed neighborhoods as evidence of reduced competition among highly related individuals. Ultimately, evidence of kin cooperation in plants is equivocal (File et al., 2012), and changes in biomass allocation can also reflect inbreeding depression (Sandner et al., 2021). Considering evidence of inbreeding depression across the life cycle in *S. angularis* (Dudash, 1990; Spigler et al., 2017; Spigler and Charles, 2023), we maintain that inbreeding depression affecting competitive ability is the most likely explanation for our results. Future experiments should incorporate treatments with unrelated selfed individuals to distinguish between inbreeding depression and genetic relatedness. Regardless, our results illustrate how the strength of density-dependence and opportunity for selection may hinge on the genetic composition of competitors.

### 4.2 Density-dependent inbreeding depression in *Sabatia angularis*

There has been a longstanding interest in understanding how inbreeding depression is influenced by environmental conditions (reviewed in Cheptou and Donohue, 2011; Sandner et al., 2021). A prevailing hypothesis is that selfed individuals, which are more homozygous, may exhibit lower tolerance to stress. Consequently, the effects of inbreeding depression should become more pronounced under stressful conditions. As Yun and Agrawal (2014) point out, while various forms of stress could exacerbate inbreeding depression, competitive stress is a particularly relevant factor to consider in this context. Our results point to inbreeding depression in traits related to resource competition rather than tolerance to competition. We saw that outcrossed individuals exert stronger competitive effects on each other when alone. Each additional competitor in an outcrossed neighborhood imposed a greater fitness burden than the addition of a selfed competitor did in selfed neighborhoods. Seen another way, we find that selfed plants benefit less from the release of competition against other selfed individuals. Consequently, outcrossed individuals in mixed neighborhoods are expected to dominate and suppress selfed ones, aligning with greater inequality observed in mixed neighborhoods. Given such inequalities increased with density, we would conclude inbreeding depression increased with density.

**Figure 3:**
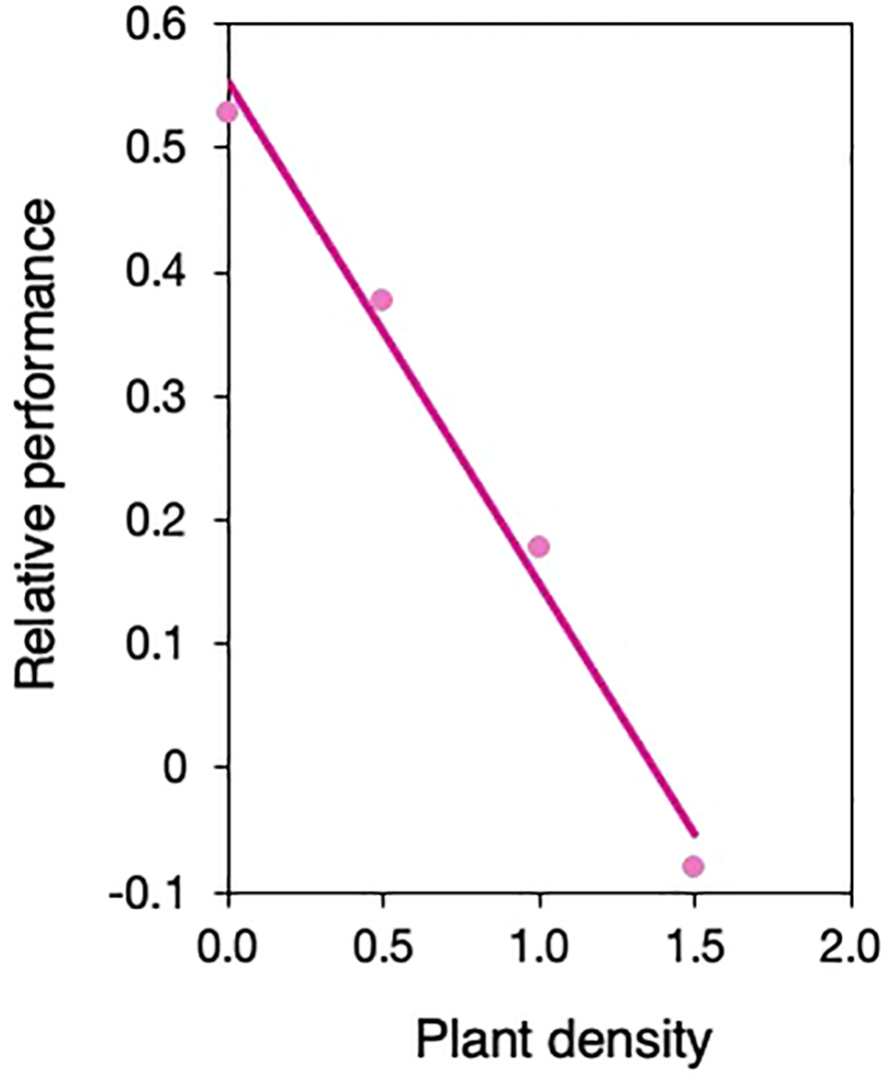
Change in relative performance of selfed vs. outcrossed neighborhoods with density. Performance is based on leaf area. Relative performance is calculated as 1-w_s_/w_o_ when *w_s_* <*w_o_* and *w_o_*/*w_s_*-1 when *w_s_* >*w_o_*, wherein *w_s_* and *w_o_* are the mean leaf area of selfed and outcrossed neighborhoods, respectively. Mean leaf area estimates are calculated from prediction lines in **Figure 1B**, determined at (log_10_-transformed) densities of 0, 0.5, 1, 1.5 using the ‘lsmestimate’ statement in proc mixed in SAS.

Yet our data also suggest that the effect of density on the relative performance of selfed vs. outcrossed individuals will depend on relationships between ecological and genetic neighborhoods and the spatial scales at which density-dependence and selection operate in wild populations (Antonovics and Levin, 1980). If individuals occur in clusters of related selfed or outcrossed individuals at high density in mixed-mating populations, inbreeding depression could decrease with density. To illustrate this, we evaluated how relative performance (as defined in Ågren and Schemske, 1993) between outcrossed and selfed neighborhoods changed with density as a *post-hoc* analysis and found a strong logarithmic decline (**Figure 3**). Others have also found larger differences in performance between monocultures of outcrossed and selfed individuals when grown alone vs. higher density (Koelewijn, 2004; Lhamo et al., 2006). All else equal (noting it rarely is), this means that selfed and outcrossed individuals could equally contribute to the next generation, maintaining mixed-mating and mutation load (but see Uyenoyama and Waller, 1991). While exclusively selfed or outcrossed clusters of plants within a population may be rare in nature, fine-scale spatial genetic structure is frequently observed in plant populations (‘FSGS’, Vekemans and Hardy, 2004), even at high densities when plants are strongly aggregated (Lara-Romero et al., 2016). This results in local intraspecific competition often occurring between related individuals. Variability in selfing rates among individuals can lead to the formation of clusters consisting of predominantly selfed or outcrossed juveniles. Although we do not have estimates of FSGS in S*. angularis* populations, we know that seeds are passively dispersed, juveniles are spatially clustered, most *S. angularis* populations are mixed mating (Spigler et al., 2010), and individual-level selfing rates vary. Preliminary calculations of family-level selfing rates using data from Spigler et al., 2010 (N=58 individuals across 3 populations) estimate that up to 10.5% of plants have high selfing rates (≥80%), while an average of 48% are outcrossing (≤20% selfing) and 45% mixed maters. Consequently, a large proportion of outcrossed juveniles likely suffer from harsher competition, while a small but potentially significant fraction of selfed juveniles could be sheltered from selection on competitive traits, hindering the purging of genetic load. We did not consider a gradient of frequencies of selfed competitors in this study but predict that inbreeding depression will also vary with selfing frequency in mixed clusters, in line with previous findings of frequency-dependence in other species (Cheptou and Schoen, 2003; Koelewijn, 2004; Lhamo et al., 2006).

Other factors can affect the extent to which the dynamics shown in our study play out in wild populations, to be addressed by future studies. On the one hand, interspecific competition could effectively harden selection, depending on the relative genetic load in competitor species and similarity of resource use (Agrawal, 2010; Agrawal and Whitlock, 2012). On the other hand, interspecific interactions may be weak if there is strong intraspecific clustering (Rees et al., 1996), and asymmetric competition may be stronger in the field (Freckleton and Watkinson, 2002). Finally, the timing and distribution of mortality has important implications for the ecological impact of soft selection (Agrawal and Whitlock, 2012). Whereas genotype-dependent mortality that occurs before resource competition can free resources, allowing fitness gains of survivors offset juvenile losses, mortality occurring after resource consumption can lead to population decline. Here, we found that survival rates throughout the juvenile growth period were similar across neighborhood types, suggesting a limited impact of mortality. However, census data from wild *S. angularis* populations also reveals high mortality in *S. angularis* after the growing season, during the overwintering period. Consequently, asymmetric competition between selfed and outcrossed individuals coupled with size-dependent overwinter survival in *S. angularis* points toward a potentially detrimental effect of soft selection on population fitness.

### 4.3 Conclusions

Our findings align with the concept of soft selection concerning inbreeding depression affecting competitive ability in *S. angularis*. Ecologically, our results imply that populations or neighborhoods at high density may exhibit comparable total yields in terms of juvenile growth, despite varying degrees of mutation loads. From an evolutionary standpoint, our findings indicate that the process of selection against selfed individuals, and consequently, the evolution of mating systems, will likely be influenced by the density and genetic makeup of ecological neighborhoods. Future work should consider later life history stages at larger spatial scales to provide deeper insights into interplay among genetic load, population dynamics, and the evolution of mating systems.

## Data Availability

The raw data supporting the conclusions of this article will be made available by the authors, without undue reservation.

## References

[B1] Abu Awad D. Billiard S. Tran V. C. (2014). The effect of the timing of selection on the mutation load, inbreeding depression and population size. Available online at: https://hal.archives-ouvertes.fr/hal-00987288 (Accessed December 22, 2022).

[B2] Addicott J. F. Aho J. M. Antolin M. F. Padilla D. K. Richardson J. S. Soluk D. A. (1987). Ecological neighborhoods: Scaling environmental patterns. Oikos 49, 340–346. doi: 10.2307/3565770

[B3] Agrawal A. F. (2010). Ecological determinants of mutation load and inbreeding depression in subdivided populations. Am. Nat. 176, 111–122. doi: 10.1086/653672 20545488

[B4] Agrawal A. F. Whitlock M. C. (2012). Mutation load: The fitness of individuals in populations where deleterious alleles are abundant. Annu. Rev. Ecol. Evol. Syst. 43, 115–135. doi: 10.1146/annurev-ecolsys-110411-160257

[B5] Ågren J. Schemske D. W. (1993). Outcrossing rate and inbreeding depression in two annual monoecious herbs, begonia hirsuta and B. Semiovata. Evolution 47, 125–135. doi: 10.2307/2410123 28568096

[B6] Antonovics J. Levin D. A. (1980). The ecological and genetic consequences of density-Dependent regulation in plants. Annu. Rev. Ecol. Syst. 11, 411–452. doi: 10.1146/annurev.es.11.110180.002211

[B7] Arenas F. Fernández C. (2000). Size structure and dynamics in a population of sargassum muticum (phaeophyceae). J. Phycol. 36, 1012–1020. doi: 10.1046/j.1529-8817.2000.99235.x

[B8] Bassar R. D. Coulson T. Travis J. Reznick D. N. (2021). Towards a more precise – and accurate – view of eco-evolution. Ecol. Lett. 24, 623–625. doi: 10.1111/ele.13712 33617684

[B9] Bell D. A. Kovach R. P. Robinson Z. L. Whiteley A. R. Reed T. E. (2021). The ecological causes and consequences of hard and soft selection. Ecol. Lett. 24, 1505–1521. doi: 10.1111/ele.13754 33931936

[B10] Brown K. E. Kelly J. K. (2020). Severe inbreeding depression is predicted by the “rare allele load” in Mimulus guttatus. Evolution 74, 587–596. doi: 10.1111/evo.13876 31710100

[B11] Brown C. Oppon K. J. Cahill J. F. Jr (2019). Species-specific size vulnerabilities in a competitive arena: Nutrient heterogeneity and soil fertility alter plant competitive size asymmetries. Funct. Ecol. 33, 1491–1503. doi: 10.1111/1365-2435.13340

[B12] Byers D. L. Waller D. M. (1999). Do plant populations purge their genetic load? Effects of population size and mating history on inbreeding depression. Annu. Rev. Ecol. Syst. 30, 479–513. doi: 10.1146/annurev.ecolsys.30.1.479

[B13] Charlesworth D. Charlesworth B. (1987). Inbreeding depression and its evolutionary consequences. Annu. Rev. Ecol. Syst. 18, 237–268. doi: 10.1146/annurev.es.18.110187.001321

[B14] Charlesworth B. Charlesworth D. (1999). The genetic basis of inbreeding depression. Genet. Res. 74, 329–340. doi: 10.1017/S0016672399004152 10689809

[B15] Charlesworth D. Willis J. H. (2009). The genetics of inbreeding depression. Nat. Rev. Genet. 10, 783–796. doi: 10.1038/nrg2664 19834483

[B16] Cheptou P.-O. Donohue K. (2011). Environment-dependent inbreeding depression: its ecological and evolutionary significance. New Phytol. 189, 395–407. doi: 10.1111/j.1469-8137.2010.03541.x 21091479

[B17] Cheptou P.-O. Lepart J. Escarré J. (2001). Inbreeding depression under intraspecific competition in a highly outcrossing population of *Crepis sancta* (Asteraceae): evidence for frequency-dependent variation. Am. J. Bot. 88, 1424–1429. doi: 10.2307/3558449 21669674

[B18] Cheptou P. Schoen D. J. (2003). Frequency-dependent inbreeding depression in Amsinckia. Am. Nat. 162, 744–753. doi: 10.1086/378902 14737712

[B19] Cheptou P. O. Schoen D. J. (2007). Combining population genetics and demographical approaches in evolutionary studies of plant mating systems. Oikos 116, 271–279. doi: 10.1111/j.0030-1299.2007.14655.x

[B20] Cook R. E. (1979). “Patterns of juvenile mortality and recruitment in plants,” in Topics in Plant Population Biology. Eds. Solbrig O. T. Jain S. Johnson G. B. Raven P. H. (Macmillan Education UK, London), 207–231.

[B21] Damgaard C. Loeschcke V. (1994). Inbreeding depression and dominance-suppression competition after inbreeding in rapeseed (*Brassica napus*). Theoret Appl. Genet. 88–88, 321–323. doi: 10.1007/BF00223639 24186013

[B22] Damgaard C. Weiner J. (2000). Describing inequality in plant size or fecundity. Ecology 81, 1139–1142. doi: 10.1890/0012-9658(2000)081[1139:DIIPSO]2.0.CO;2

[B23] Dudash M. R. (1990). Relative fitness of selfed and outcrossed progeny in a self-compatible, protandrous species, *Sabatia angularis* L. (gentianaceae): a comparison in three environments. Evolution 44, 1129–1139. doi: 10.2307/2409277 28563899

[B24] Dudash M. R. (1991). Plant size effects on female and male function in hermaphroditic *Sabatia angularis* (Gentianaceae). Ecology 72, 1004–1012. doi: 10.2307/1940600

[B25] Dudley S. A. File A. L. (2007). Kin recognition in an annual plant. Biol. Lett. 3, 435–438. doi: 10.1098/rsbl.2007.0232 17567552 PMC2104794

[B26] Ehlers B. K. Bilde T. (2019). Inclusive fitness, asymmetric competition and kin selection in plants. Oikos 128, 765–774. doi: 10.1111/oik.06390

[B27] File A. L. Murphy G. P. Dudley S. A. (2012). Fitness consequences of plants growing with siblings: reconciling kin selection, niche partitioning and competitive ability. Proc. R. Soc. B: Biol. Sci. 279, 209–218. doi: 10.1098/rspb.2011.1995 PMC3223689 22072602

[B28] Fox C. Reed D. (2011). Inbreeding depression increases with environmental stress: an experimental study and meta-analysis. Evolution 65, 246–258. doi: 10.1111/j.1558-5646.2010.01108.x 20731715

[B29] Freckleton R. P. Watkinson A. R. (2002). Are weed population dynamics chaotic? J. Appl. Ecol. 39, 699–707. doi: 10.1046/j.1365-2664.2002.00748.x

[B30] Gervais C. Awad D. A. Roze D. Castric V. Billiard S. (2014). Genetic architecture of inbreeding depression and the maintenance of gametophytic self-incompatibility. Evolution 68, 3317–3324. doi: 10.1111/evo.12495 25065256

[B31] Goodwillie C. Kalisz S. Eckert C. G. (2005). The evolutionary enigma of mixed mating systems in plants: Occurrence, theoretical explanations, and empirical evidence. Annu. Rev. Ecol. Evol. Syst. 36, 47–79. doi: 10.1146/annurev.ecolsys.36.091704.175539

[B32] Harper J. L. (1977). Population Biology of Plants (New York, NY: Academic Press).

[B33] Ho E. K. H. Agrawal A. F. (2012). The effects of competition on the strength and softness of selection. J. Evol. Biol. 25, 2537–2546. doi: 10.1111/j.1420-9101.2012.02618.x 23020134

[B34] Holsinger K. E. Pacala S. W. (1990). Multiple-niche polymorphisms in plant populations. Am. Nat. 135, 301–309. doi: 10.1086/285046

[B35] Horvitz C. C. Schemske D. W. (1995). Spatiotemporal variation in demographic transitions of a tropical understory herb: projection matrix analysis. Ecol. Monogr. 65, 155–192. doi: 10.2307/2937136

[B36] Husband B. C. Schemske D. W. (1996). Evolution of the magnitude and timing of inbreeding depression in plants. Evolution 50, 54–70. doi: 10.2307/2410780 28568860

[B37] Jarne P. Charlesworth D. (1993). The Evolution of the selfing rate in functionally hermaphrodite plants and animals. Annu. Rev. Ecol. Syst. 24, 441–466. doi: 10.1146/annurev.es.24.110193.002301

[B38] Keller L. F. Waller D. M. (2002). Inbreeding effects in wild populations. Trends Ecol. Evol. 17, 230–241. doi: 10.1016/S0169-5347(02)02489-8

[B39] Koelewijn H. P. (2004). Sibling competition, size variation and frequency-dependent outcrossing advantage in *Plantago coronopus* . Evol. Ecol. 18, 51–74. doi: 10.1023/B:EVEC.0000017695.64459.e3

[B40] Kokko H. López-Sepulcre A. (2007). The ecogenetic link between demography and evolution: can we bridge the gap between theory and data? Ecol. Lett. 10, 773–782. doi: 10.1111/j.1461-0248.2007.01086.x 17663710

[B41] Laffafian A. King J. D. Agrawal A. F. (2010). Variation in the strength and softness of selection on deleterious mutations. Evolution 64, 3232–3241. doi: 10.1111/evo.2010.64.issue-11 20662923

[B42] Lande R. (1982). A quantitative genetic theory of life history evolution. Ecology 63, 607–615. doi: 10.2307/1936778

[B43] Lande R. Schemske D. W. (1985). The evolution of self-fertilization and inbreeding depression in plants. I. Genetic Models. Evolution. 39 (1), 24–40. doi: 10.1111/j.1558-5646.1985.tb04077.x 28563655

[B44] Lara-Romero C. García-Fernández A. Robledo-Arnuncio J. J. Roumet M. Morente-López J. López-Gil A.. (2016). Individual spatial aggregation correlates with between-population variation in fine-scale genetic structure of *Silene ciliata* (Caryophyllaceae). Heredity 116, 417–423. doi: 10.1038/hdy.2015.102 26604191 PMC4834382

[B45] Lhamo N. Ramsey M. Vaughton G. (2006). Density- and frequency-dependent inbreeding depression in the Australian annual Hibiscus trionum var. *vesicarius* . Evol. Ecol. Res. 8, 717–730.

[B46] Loveless M. D. Hamrick J. L. (1984). Ecological determinants of genetic structure in plant populations. Annu. Rev. Ecol. Systematics 15, 65–95. doi: 10.1146/annurev.es.15.110184.000433

[B47] Metcalf C. J. E. Pavard S. (2007). All paths to fitness lead through demography. Trends Ecol. Evol. 22, 563–564. doi: 10.1016/j.tree.2007.07.003 17174004

[B48] Purves D. W. Law P. (2002). Fine-scale spatial structure in a grassland community: quantifying the plant’s eye view. J. Ecol. 90, 121–129. doi: 10.1046/j.0022-0477.2001.00652.x

[B49] Ramula S. Buckley Y. M. (2009). Multiple life stages with multiple replicated density levels are required to estimate density dependence for plants. Oikos 118, 1164–1173. doi: 10.1111/j.1600-0706.2009.17595.x

[B50] Rasmussen C. R. Weisbach A. N. Thorup-Kristensen K. Weiner J. (2019). Size-asymmetric root competition in deep, nutrient-poor soil. J. Plant Ecol. 12, 78–88. doi: 10.1093/jpe/rtx064

[B51] Rees M. Grubb P. J. Kelly D. (1996). Quantifying the impact of competition and spatial heterogeneity on the structure and dynamics of a four-species guild of winter annuals. Am. Nat. 147, 1–32. doi: 10.1086/285837

[B52] Sandner T. M. Matthies D. Waller D. M. (2021). Stresses affect inbreeding depression in complex ways: disentangling stress-specific genetic effects from effects of initial size in plants. Heredity 127, 347–356. doi: 10.1038/s41437-021-00454-5 34188195 PMC8478953

[B53] Schmidt K. P. Lawlor L. R. (1983). Growth rate projection and life history sensitivity for annual plants with a seed bank. Am. Nat. 121, 525–539. doi: 10.1086/284080

[B54] Schmitt J. Ehrhardt D. W. (1990). Enhancement of inbreeding depression by dominance and suppression in *Impatiens capensis* . Evolution 44, 269–278. doi: 10.2307/2409406 28564389

[B55] Schneider C. A. Rasband W. S. Eliceiri K. W. (2012). NIH Image to ImageJ: 25 years of image analysis. Nat. Methods 9, 671–675. doi: 10.1038/nmeth.2089 22930834 PMC5554542

[B56] Schwinning S. Weiner J. (1998). Mechanisms determining the degree of asymmetry in competition among plants. Oecologia 113, 447–455. doi: 10.1007/s004420050397 28308024

[B57] Shefferson R. P. Salguero-Gómez R. (2015). Eco-evolutionary dynamics in plants: Interactive processes at overlapping time-scales and their implications. J. Ecol. 103 (4), 789–797. doi: 10.1111/1365-2745.12432

[B58] Silvertown J. Charlesworth D. (2001). ntroduction to plant population biology, fourth edition (Oxford and Malden, Massachusetts: Blackwell Science).

[B59] Spigler R. B. (2017). Plasticity of floral longevity and floral display in the self-compatible biennial *Sabatia angularis* (Gentianaceae): untangling the role of multiple components of pollination. Ann. Bot. 119, 167–176. doi: 10.1093/aob/mcw195 28062510 PMC5218385

[B60] Spigler R. B. (2018). Small and surrounded: population size and land use intensity interact to determine reliance on autonomous selfing in a monocarpic plant. Ann. Bot. 121, 513–524. doi: 10.1093/aob/mcx184 29346506 PMC5838805

[B61] Spigler R. B. Charles A. (2023). Inbreeding reduces floral longevity and flower size in the mixed-mating biennial *Sabatia angularis* . Int. J. Plant Sci. 184, 157–163. doi: 10.1086/724030

[B62] Spigler R. B. Hamrick J. L. Chang S.-M. (2010). Increased inbreeding but not homozygosity in small populations of *Sabatia angularis* (Gentianaceae). Plant Syst. Evol. 284, 131–140. doi: 10.1007/s00606-009-0245-x

[B63] Spigler R. B. Theodorou K. Chang S.-M. (2017). Inbreeding depression and drift load in small populations at demographic disequilibrium. Evolution 71, 81–94. doi: 10.1111/evo.13103 27778313

[B64] Theodorou K. Couvet D. (2006). On the expected relationship between inbreeding, fitness, and extinction. Genet. Sel. Evol. 38, 371–387. doi: 10.1186/1297-9686-38-4-371 16790228 PMC2689291

[B65] Thomas S. C. Weiner J. (1989). Including competitive asymmetry in measures of local interference in plant populations. Oecologia 80, 349–355. doi: 10.1007/BF00379036 28312062

[B66] Uyenoyama M. K. Waller D. M. (1991). Coevolution of self-fertilization and inbreeding depression I. Mutation-selection balance at one and two loci. Theor. Population Biol. 40, 14–46. doi: 10.1016/0040-5809(91)90045-H 1948770

[B67] Vekemans X. Hardy O. J. (2004). New insights from fine-scale spatial genetic structure analyses in plant populations. Mol. Ecol. 13, 921–935. doi: 10.1046/j.1365-294X.2004.02076.x 15012766

[B68] Walker M. J. (2023). Resource Competition and Soft Selection in *Sabatia angularis* (United States – Pennsylvania: Temple University). Available online at: https://www.proquest.com/docview/2912094918/abstract/43CF552FAFCE4E9DPQ/1 (Accessed Mar 3, 2024).

[B69] Wallace B. (1975). Hard and soft selection revisited. Evolution 29, 465–473. doi: 10.2307/2407259 28563194

[B70] Waller D. M. (1985). The genesis of size hierarchies in seedling populations of *Impatiens capensis* Meerb. New Phytol. 100, 243–260. doi: 10.1111/j.1469-8137.1985.tb02776.x

[B71] Waller D. M. (2021). Addressing Darwin’s dilemma: Can pseudo-overdominance explain persistent inbreeding depression and load? Evolution 75, 779–793. doi: 10.1111/evo.14189 33598971

[B72] Weiner J. (1985). Size hierarchies in experimental populations of annual plants. Ecology 66, 743–752. doi: 10.2307/1940535

[B73] Weiner J. (1990). Asymmetric competition in plant populations. Trends Ecol. Evol. 5, 360–364. doi: 10.1016/0169-5347(90)90095-U 21232393

[B74] Weiner J. Damgaard C. (2006). Size-asymmetric competition and size-asymmetric growth in a spatially explicit zone-of-influence model of plant competition. Ecol. Res. 21, 707–712. doi: 10.1007/s11284-006-0178-6

[B75] Weiner J. Freckleton R. P. (2010). Constant final yield. Annu. Rev. Ecol. Evol. Syst. 41, 173–192. doi: 10.1146/annurev-ecolsys-102209-144642

[B76] Weiner J. Solbrig O. T. (1984). The meaning and measurement of size hierarchies in plant populations. Oecologia 61, 334–336. doi: 10.1007/BF00379630 28311058

[B77] Weiner J. Thomas S. C. (1986). Size variability and competition in plant monocultures. Oikos 47, 211–222. doi: 10.2307/3566048

[B78] Weis A. E. Turner K. M. Petro B. Austen E. J. Wadgymar S. M. (2015). Hard and soft selection on phenology through seasonal shifts in the general and social environments: A study on plant emergence time. Evolution 69, 1361–1374. doi: 10.1111/evo.12677 25929822

[B79] Wennerberg S. B. (2005). Propagation and field assessment of West Virginia native species for roadside revegetation. Graduate Theses, Dissertations, and Problem Reports. 2207. West Virginia, USA: West Virginia University. Available at: https://researchrepository.wvu.edu/etd/2207.

[B80] Whitlock M. C. (2002). Selection, load and inbreeding depression in a large metapopulation. Genetics 160, 1191–1202. doi: 10.1093/genetics/160.3.1191 11901133 PMC1462034

[B81] Wickham H. (2016). ggplot2: Elegant graphics for data analysis (New York: Springer-Verlag). Available at: https://ggplot2.tidyverse.org.

[B82] Winn A. A. Elle E. Kalisz S. Cheptou P. O. Eckert C. G. Goodwillie C.. (2011). Analysis of inbreeding depression in mixed-mating plants provides evidence for selective interference and stable mixed mating. Evolution 65, 3339–3359. doi: 10.1111/evo.2011.65.issue-12 22133210

[B83] Wolfe L. M. (1993). Inbreeding depression in *Hydrophyllum appendiculatum*: role of maternal effects, crowding, and parental mating history. Evolution 47, 374–386. doi: 10.2307/2410058 28568732

[B84] Yun L. Agrawal A. F. (2014). Variation in the strength of inbreeding depression across environments: Effects of stress and density dependence. Evolution 68, 3599–3606. doi: 10.1111/evo.12527 25213285

[B85] Zeileis A. Kleiber C. (2014). ineq: Measuring Inequality, Concentration, and Poverty. Available online at: https://cran.r-project.org/web/packages/ineq/index.html (Accessed October 12, 2022).

