# Supplementary material for: Experimental evidence of inbreeding depression for competitive ability and its population-level consequences in a mixed-mating plant
Source: Front Plant Sci. 2024 Sep 17;15:1398060. doi: 10.3389/fpls.2024.1398060 (PMC11442323; doi:10.3389/fpls.2024.1398060)
Supplement: Supplementary file 1 [file DataSheet1.docx]

**Supplemental Information**

Experimental evidence of inbreeding depression for competitive ability and its population-level consequences in a mixed-mating plant.

MJ Walker & RB Spigler


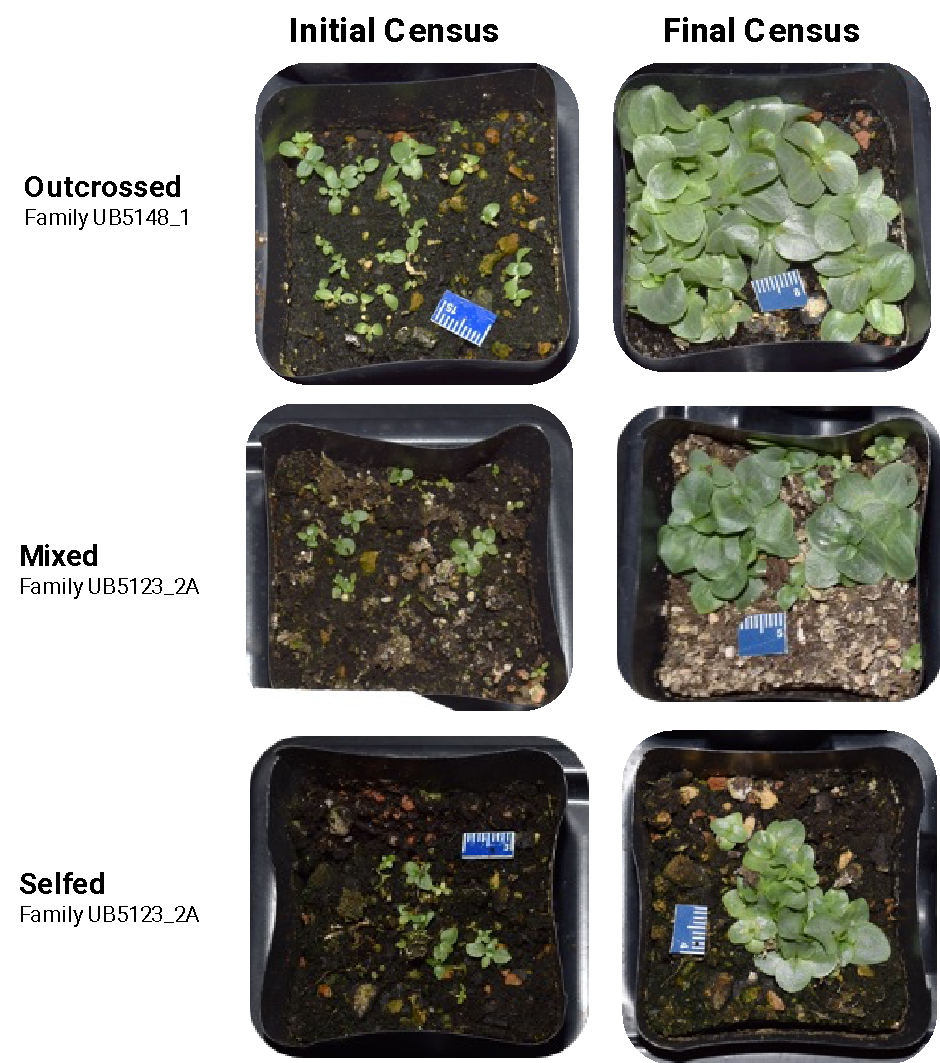


**Supplementary Figure S1.** Examples of competitive arenas illustrating size and growth of *Sabatia angularis* for three neighborhood compositions (completely outcrossed, mixed, and completely selfed) at initial and final censuses.

**Supplementary Table S1.** Sample sizes.

**Table S1a.** Sample sizes by family, planting density, and neighborhood composition.

**Table S1b.** Summary of sample sizes for each stage of the study by family and neighborhood composition.

Notes: M=mixed, O=outcrossed, S=selfed neighborhoods (pots)

**Supplemental Table S2.** Full model results for germination rate including a single influential data point and accompanying post hoc statistical tests.

| **Fixed effects** | **Num df** | **Den df** | ***F*** | ***P*** |
| --- | --- | --- | --- | --- |
| Neighborhood composition | 2 | 175 | 1.34 | 0.26 |
| Planting (seed) density | 1 | 175 | 4.38 | 0.04 |
| Neighborhood ｘ Planting density | 2 | 175 | 4.78 | 0.01 |
| **Random effects** |  |  | ***Z*** | ***P*** |
| Tray |  |  | 2 | 0.02 |
| Family |  |  | 0.04 | 0.48 |

| **Label** | **Estimate** | **Standard Error** | **DF** | **t Value** | **Pr > \|t\|** |
| --- | --- | --- | --- | --- | --- |
| slope, Mixed | -2.90E-04 | 9.10E-05 | 175 | -3.15 | 0.002 |
| slope, Outcrossed | -1.20E-04 | 9.90E-05 | 175 | -1.17 | 0.24 |
| slope, Selfed | 7.70E-05 | 7.50E-05 | 175 | 1.02 | 0.31 |
| diff slopes, O-M | -0.176 | 0.083 | 1583 | -2.13 | 0.03 |
| diff slopes, O-S | 0.0788 | 0.078 | 1583 | 1.01 | 0.31 |
| diff slopes, M-S | 0.2543 | 0.087 | 1583 | 2.92 | 0.004 |

Notes: O=outcrossed, M=mixed, S=selfed

**Supplemental Table S3.** Estimates of the slope quantifying the relationship between juvenile size (log_10_-transformed) and juvenile density (log_10_-transformed) at the initial census and accompanying pairwise tests for significant differences in slopes across neighborhood composition treatments.

| **Label** | **Estimate** | **Standard Error** | **DF** | **t Value** | **Pr > \|t\|** | ***P_adj_*** |
| --- | --- | --- | --- | --- | --- | --- |
| slope, Mixed | 0.227 | 0.064 | 1583 | 3.54 | 0.0004 | 0.001 |
| slope, Outcrossed | 0.052 | 0.052 | 1583 | 1 | 0.32 | 0.95 |
| slope, Selfed | -0.027 | 0.059 | 1583 | -0.46 | 0.65 | 1.00 |
| diff slopes, O-M | -0.176 | 0.083 | 1583 | -2.13 | 0.0335 | 0.10 |
| diff slopes, O-S | 0.0788 | 0.078 | 1583 | 1.01 | 0.3133 | 0.94 |
| diff slopes, M-S | 0.2543 | 0.087 | 1583 | 2.92 | 0.0036 | 0.01 |

Notes: O=outcrossed, M=mixed, S=selfed

**Supplemental Table S4.** Tests for differences in juvenile size (log_10_-transformed) across neighborhood composition treatments at three densities (log_10_-transformed) at the initial census. Estimated at mean median date of germination. (O=outcrossed, M=mixed, S=selfed)

| **Contrast Pair**  **Neighborhood** | | **Density**  **(**log_10_**)** | **Estimate** | **Standard**  **Error** | **DF** | **t** | **Pr > \|t\|** | ***P_adj_*** |
| --- | --- | --- | --- | --- | --- | --- | --- | --- |
| M | O | 0 | -0.342 | 0.117 | 34 | -2.93 | 0.006 | 0.016 |
| M | S | 0 | -0.343 | 0.124 | 34 | -2.78 | 0.009 | 0.023 |
| O | S | 0 | -0.002 | 0.115 | 34 | -0.01 | 0.98 | 0.99 |
| M | O | 0.5 | -0.254 | 0.086 | 34 | -2.96 | 0.006 | 0.015 |
| M | S | 0.5 | -0.216 | 0.090 | 34 | -2.4 | 0.02 | 0.056 |
| O | S | 0.5 | 0.0377 | 0.084 | 34 | 0.45 | 0.66 | 0.90 |
| M | O | 1 | -0.166 | 0.067 | 34 | -2.47 | 0.02 | 0.05 |
| M | S | 1 | -0.089 | 0.069 | 34 | -1.29 | 0.21 | 0.41 |
| O | S | 1 | 0.077 | 0.064 | 34 | 1.2 | 0.24 | 0.46 |
| M | O | 2 | 0.009 | 0.095 | 34 | 0.1 | 0.92 | 0.99 |
| M | S | 2 | 0.165 | 0.097 | 34 | 1.7 | 0.10 | 0.22 |
| O | S | 2 | 0.156 | 0.086 | 34 | 1.82 | 0.08 | 0.18 |

**Supplemental Table S5.** Estimates of the slope quantifying the relationship between juvenile size (log_10_-transformed) and juvenile density (log_10_-transformed) at the final census and accompanying pairwise tests for significant differences in slopes across neighborhood composition treatments.

| **Label** | **Estimate** | **Standard Error** | **DF** | **t Value** | **Pr > \|t\|** | ***P_adj_*** |
| --- | --- | --- | --- | --- | --- | --- |
| Slope, Mixed | -0.248 | 0.06685 | 1302 | -3.71 | 0.0002 | 0.0006 |
| Slope, Outcrossed | -0.600 | 0.04743 | 1302 | -12.64 | <.0001 | <0.0001 |
| Slope, Selfed | -0.359 | 0.04968 | 1302 | -7.23 | <.0001 | <0.0001 |
| diff slopes, O-M | -0.351 | 0.082 | 1302 | -4.30 | <0.0001 | <0.0001 |
| diff slopes, O-S | -0.241 | 0.068 | 1302 | -3.53 | 0.0004 | 0.001 |
| diff slopes, M-S | 0.111 | 0.083 | 1302 | 1.34 | 0.18 | 0.54 |

**Supplemental Table S6.** Tests for differences in juvenile size (log_10_-transformed) across neighborhood composition treatments at three densities (log_10_-transformed) at the final census. Estimated at mean median date of germination.

| **Neighborhood Contrast Pair** | | **Density (**log_10_**)** | **Estimate** | **Standard Error** | **DF** | ***t*** | **Pr>\|t\|** | ***P_adj_*** |
| --- | --- | --- | --- | --- | --- | --- | --- | --- |
| M | O | 0 | -0.479 | 0.099 | 36 | -4.83 | <0.0001 | <0.0001 |
| M | S | 0 | -0.154 | 0.100 | 36 | -1.54 | 0.13 | 0.29 |
| O | S | 0 | 0.325 | 0.089 | 36 | 3.67 | 0.001 | 0.002 |
| M | O | 0.5 | -0.303 | 0.072 | 36 | -4.23 | 0.0002 | 0.0004 |
| M | S | 0.5 | -0.099 | 0.072 | 36 | -1.37 | 0.18 | 0.37 |
| O | S | 0.5 | 0.205 | 0.066 | 36 | 3.12 | 0.004 | 0.01 |
| M | O | 1 | -0.128 | 0.062 | 36 | -2.08 | 0.05 | 0.11 |
| M | S | 1 | -0.043 | 0.062 | 36 | -0.7 | 0.49 | 0.76 |
| O | S | 1 | 0.084 | 0.056 | 36 | 1.52 | 0.14 | 0.3 |
| M | O | 2 | 0.224 | 0.105 | 36 | 2.13 | 0.04 | 0.1 |
| M | S | 2 | 0.068 | 0.106 | 36 | 0.64 | 0.53 | 0.8 |
| O | S | 2 | -0.156 | 0.087 | 36 | -1.79 | 0.08 | 0.19 |

Note: O=outcrossed, M=mixed, S=selfed

**Supplemental Table S7**. Model results for size inequality, estimated as the Gini Coefficient, including 2 influential data points, with and without the non-significant interaction term.

|  | **With interaction** | | | |  | **Without interaction** | | | |
| --- | --- | --- | --- | --- | --- | --- | --- | --- | --- |
| **Fixed effects** | **Num  df** | **Den  df** | ***F*** | ***P*** |  | **Num  df** | **Den  df** | ***F*** | ***P*** |
| Neighborhood type  (mixed vs. homogeneous) | 1 | 50 | 1.01 | 0.32 |  | 1 | 51 | 4.29 | 0.04 |
| Density | 1 | 50 | 6.03 | **0.02** |  | 1 | 51 | 10.1 | **0.003** |
| Neighborhood ｘ Density | 1 | 50 | 0.29 | 0.59 |  | -- | -- | -- | -- |
| **Random effects** |  |  | ***Z*** | ***P*** |  |  |  | ***Z*** | ***P*** |
| Family |  |  | 0.75 | 0.23 |  |  |  | 0.85 | 0.2 |
| **Pairwise contrasts** |  | **df** | ***t*** | ***P*** |  |  | **df** | ***t*** | ***P*** |
| Mixed vs. homogeneous |  | 50 | 2.04 | **0.05** |  |  | 51 | 2.07 | **0.04** |
